# Potential Application of Nitrogen-Doped Carbon Quantum Dots Synthesized by a Solvothermal Method for Detecting Silver Ions in Food Packaging

**DOI:** 10.3390/ijerph16142518

**Published:** 2019-07-14

**Authors:** Zifan Lu, Tiantian Su, Yanting Feng, Shiqi Jiang, Chunxia Zhou, Pengzhi Hong, Shengli Sun, Chengyong Li

**Affiliations:** 1 College of Food Science and Technology, Guangdong Ocean University, Zhanjiang 524088, China; 2 Shenzhen Institute of Guangdong Ocean University, Shenzhen 518108, China; 3 School of Chemistry and Environment, Guangdong Ocean University, Zhanjiang 524088, China

**Keywords:** Nitrogen-doped carbon quantum dots, fluorescence intensity, Ag^+^, food packaging material

## Abstract

In this paper, nitrogen-doped carbon quantum dots (N-CQDs) were synthesized by a solvothermal method using 1,2,4-triaminobenzene as a carbon precursor. The surface of the synthesized N-CQDs was modified with amino functional groups. The results indicated that N-CQDs had various N-related functional groups and chemical bonds and were amorphous in structure. At the same time, the quantum yield of N-CQDs was 5.11%, and the average lifetime of fluorescence decay was 5.79 ns. The synthesized N-CQDs showed good selectivity for and sensitivity to Ag^+^. A linear relationship between N-CQDs detection efficiency and Ag^+^ concentration was observed for concentration ranges of Ag^+^ corresponding to 0–10 μM and 10–30 μM. In addition, N-CQDs were used for the detection of trace Ag^+^ in food packaging material. The silver ion content of the sample determined by the N-CQDs detection method was 1.442 mg/L, with a relative error of 6.24% with respect to flame atomic absorption spectrometry, according to which the Ag^+^ content was 1.352 mg/L. This indicates that the N-CQDs detection method is reliable. Therefore, the N-CQDs prepared in this paper can detect Ag^+^ rapidly, simply, and sensitively and are expected to be a promising tool for the detection of trace Ag^+^ in food packaging materials.

## 1. Introduction

It is well known that silver ions have a bactericidal action. The bactericidal mechanism of silver ions is related to the strong interaction of ionic silver with the sulfhydryl group of enzymes, which results in enzyme inactivation [1,2,3], enhanced caspase-3 activity, and DNA ladder formation in mammalian cells [4]. Small electron-dense granules formed by silver and sulfur cause changes in cell membrane structure and production of free radical [5]. In the research of food packaging material, materials containing silver nanoparticles (AgNPs) for food packaging are classified into non-degradable polymer matrices [6,7,8] and biodegradable coating films made of a polymer or a stabilizer [9,10,11]. The addition of AgNPs as an antimicrobial agent in food packaging material is a mature technology, but there is still a risk of safety accidents caused by the migration of silver ions (Ag^+^) from the package to food. Numerous studies have shown that AgNPs have a toxic effect on the skin, liver, lung, brain, and vascular cells of mammals [12,13,14,15]. It has been reported that AgNPs may induce cytotoxicity in phagocytic cells (such as mouse peritoneal macrophages) and human monocytes. It has been suggested that cytotoxicity can be induced by reactive oxygen species (ROS) at low concentrations and for short incubation times, resulting in cellular apoptosis [16,17,18,19,20]. Silver is highly toxic to various microorganisms [21]. Studies have shown that DNA loses its ability to replicate, and the structure of the cell membrane changes after treatment with silver ions [5].

Conventional methods widely used to detect silver ions include atomic absorption spectrometry (AAS) [22] and inductively coupled plasma mass spectrometry (ICP–MS) [23]. However, complex instrumentation and sample preparation processes limit the application of AAS and ICP–MS for silver ion detection. As a common metal element analysis method, flame atomic absorption spectrometry (FAAS) has a wide range of applications due to its simple analytical operation and fast analysis. However, this method is limited by the low atomization efficiency and short residence time of the atomic vapor in the optical path [24]. Electrothermal atomic absorption spectrometry (ETAAS) is a method with the advantages of high sensitivity and low cost [25]. However, it is limited by the lack of certification references for trace metals in different materials. Fluorescent sensors are applied in various biological and environmental matrices for the detection of metal ions at ng or sub-ng levels due to their high simplicity and sensitivity [26,27,28].

In recent years, carbon-based fluorescent nanomaterials have attracted great attention for the development of molecular diagnostic tools [29]. Carbon quantum dots (CQDs) have been widely used in photovoltaic devices [30,31] and photocatalysis [32] because of their superior luminescence, good biocompatibility [33], and strong resistance to photodegradation [34,35]. Element doping increases quantum yield greatly. Nitrogen-doped carbon quantum dots (N-CQDs) exhibit outstanding performance in bioimaging and catalysis [36,37]. It has been reported that N-CQDs have high quantum yields and a large number of semiconductor quantum dots, and the emission intensity of N-CQDs depends on their nitrogen content [38,39,40,41].

In this paper, N-CQDs with yellow-green fluorescence were prepared by surface functionalization of carbon quantum dots through a solvent-thermal reaction. N-CQDs were used for the detection of trace Ag^+^, demonstrating that they provide a new way for the detection of trace Ag^+^ in food packaging materials.

## 2. Materials and Methods

### 2.1. Materials

The compound 1,2,4-triaminobenzene was purchased from Wuhan Belka Biomedical Co., Ltd. (Wuhan, China). Methanol and nitric acid were from Guangdong Guanghua Sci-Tech Co., Ltd. (Guangdong, China). Formamide was from Shandong Xiya Reagent Co., Ltd. (Shandong, China). Methylene chloride was from Sinopharm Chemical Reagent Co., Ltd. (Shanghai, China). Column Chromatography Silica Gel 200–300 mesh was from Qingdao Ocean Chemical Co., Ltd. (Qingdao, China). All reagent were of analytical grade. Ultrapure water (Milli-Q, Merck, Darmstadt, Germany) was used for the preparation of the solutions. The food storage box was purchased from Yiwu Ouxing E-Commerce Co., Ltd. (Yiwu, China).

### 2.2. Instrumentation

Transmission electron microscope (TEM) images were collected from a Tecnai G2 20 transmission electron microscope (FEI company, USA). A transient fluorescence spectrometer (FLS 980, Edinburgh Instruments, UK) was used for analysis of fluorescence lifetime. A fluorescence spectrophotometer (F-7000, Hitachi High-Tech, Japan) was used for fluorescence intensity detection. An atomic absorption spectrometer (TAS990AFG, Beijing Purkinje General, China) was used to detect Ag^+^ by the Chinese standard method GB 11907-89. The characterization and structure of N-CQDs were measured by Fourier-transform infrared spectroscopy (FT-IR: TENSOR-27, Bruker, Germany). X-ray photoelectron spectroscopy (XPS) measurements were recorded using an ESCALAB 250XI (Thermo Fisher Scientific, USA). Elemental analysis was carried out by an Organic Elemental Analyzer (Elementar Vario EL Ⅲ, Elementar, Germany).

### 2.3. Synthesis of Nitrogen-Doped Carbon Quantum Dots

First, 100 mg of 1,2,4- triaminobenzene was dissolved in 10 mL of formamide, then the mixture was transferred to a Teflon-lined autoclave chamber and heated at 120 °C for 12 h. After that, the autoclave was cooled to room temperature in air atmosphere. The reaction mixture was centrifuged at 10,000 rpm for 10 min to remove large particles. Finally, the aqueous solution was purified by silica gel column chromatography using a mixture of dichloromethane and methanol (2:8 v/v) as the eluent. The eluted mixture was condensed in a rotating evaporator to obtain the purified N-CQDs.

### 2.4. Determination of ϕ

A correlation method was used to determine the fluorescence quantum yield (QY) of N-CQDs. It worth noting that the QY of rhodamine 6G (QY = 95% in ethanol) was regarded as a reference. The QY of the sample was calculated by formula (1):

(1) $$\varphi =\varphi^{\prime }\times \frac{A^{\prime }}{I^{\prime }}\times \frac{I}{A}\times \frac{n^{2}}{{n^{\prime }}^{2}}$$

where *φ* represents the quantum yield of the sample, *φ′* is the quantum yield of rhodamine’s 6G, *I* is the comprehensive emissions intensity of the tested sample, *I′* is the comprehensive emission intensity of rhodamine 6G, *n* represents the refractive index of the tested sample (1.33 for water, 1.36 for ethanol), *n′* is the refraction index of rhodamine 6G, *A* is the optical density of the tested sample, and *A′* is the optical density of rhodamine 6G. Absorption at excitation wavelength was always maintained below 0.05 to minimize the resorption effect.

### 2.5. Determination of Fluorescence Lifetime

The fluorescence lifetime of quantum dots was fitted by formula (2), and the weighted-average fluorescence lifetime $\tau_{\mathrm{av}}$ was calculated by formula (3):

(2) $$R(t)=B_{1}e^{\left(-\frac{t}{\tau_{1}}\right)}+B_{2}e^{\left(-\frac{t}{\tau_{2}}\right)}\;$$

(3) $$\tau_{\mathrm{av}}=\frac{\sum B_{i}\tau_{i}^{2}}{\sum B_{i}\tau_{i}}\;$$

where τ_1_ and τ_2_ represent time constants, and B_1_ and B_2_ represent the weight.

### 2.6. Fluorescence Intensity of N-CQDs at Different Dilutions

The prepared N-CQDs were diluted 10, 20, 50, 60, 100, 200, and 500 times by ultrapure water, and the fluorescence intensity was measured by a fluorescence spectrometer. All fluorescence spectra had an excitation wavelength of 400 nm and an emission intensity of 546 nm.

### 2.7. Determination of Ag^+^ Concentration in an Aqueous Solution by N-CQDs

The performance in the detection of Ag^+^ in an aqueous solution was evaluated by fluorescence. Different concentrations of Ag^+^ (0–30 μM) were added to the N-CQDs solutions (pH = 7), and the fluorescence was measured after reaction for 2 min.

### 2.8. Effect of Different Metal Ions on the Fluorescence Intensity of N-CQDs

Ag^+^, Hg^2+^, Cu^2+^, Cd^2+^, Pb^2+^, Co^2+^, Zn^2+^, Mn^2+^ were selected to explore the selectivity of N-CQDs. Metal ions with a concentration of 25 μM were added separately to N-CQDs solutions (pH = 7). Fluorescence was measured after reaction for 2 min. Cadmium selenide (CdSe) quantum dots containing no nitrogen were selected as a reference [42].

### 2.9. Detection of Ag^+^ in Food Packaging Materials

First, 3.0–4.0 g of food was transferred from the preservation box to a muffle furnace for melting at 450 °C for 4 h. First, 3.0–4.0 g of food preservation box was transferred to a muffle furnace for melting at 450 °C for 4 hours. Then, the melted samples were cooled to room temperature, and 2 mL of nitric acid was added to wet the samples. After that, each mixture was steamed until almost dry to remove the acid, and the dry samples were washed several times with 50 mL of ultrapure water. Subsequently, 2 mL of the sample solutions and 3 mL of N-CQDs were put into a colorimetric tube, followed by the addition of NaOH (0.5 mol/L) to adjust the pH to 7.0. Afterward, the fluorescence intensity was measured.

In total, 3.0 g of sample was weighed for dry incineration, and parallel experiments were carried out. The content of Ag^+^ in the samples was determined by the Chinese standard method GB 11907-89.

## 3. Results and Discussion

**Figure 1:**
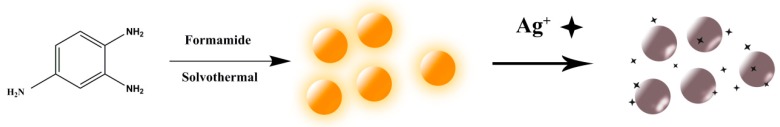
Mechanism of silver ions detection by nitrogen-doped carbon quantum dots (N-CQDs).

The mechanism of silver ions detection by N-CQDs is shown in Figure 1. When N-CQDs react with Ag^+^, the fluorescence of N-CQDs is quenched. Fluorescence quenching of N-CQDs can be attributed to the interaction of Ag^+^ and N-CQDs, which promotes a transfer of charge from the excited state of N-CQDs to Ag^+^.

### 3.1. Characterization of Structure and Composition

**Figure 2:**
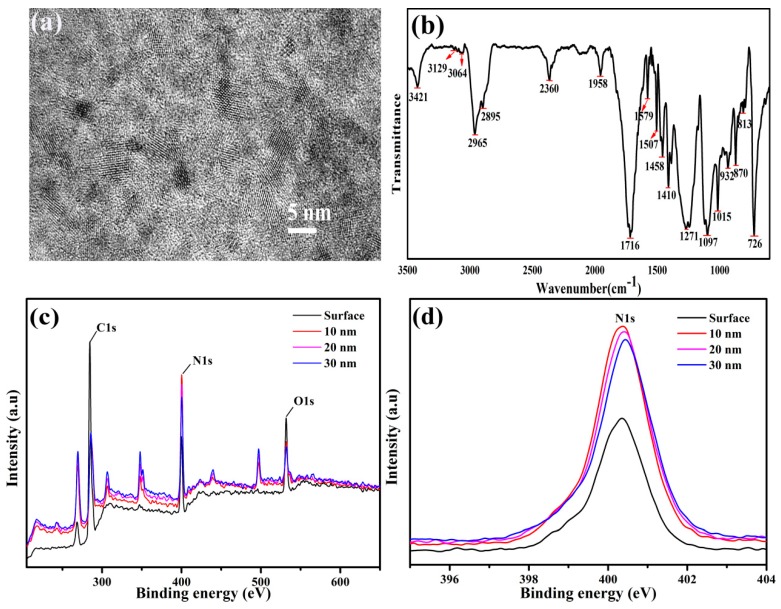
Characterization of N-CQDs. (**a**) TEM image of N-CQDs; (**b**) FT-IR spectrum of N-CQDs; (**c**) depth profiles of N-CQDs XPS spectra; (**d**) N1s high-resolution XPS spectra.

First, the prepared N-CQDs were tested with high-resolution TEM. As shown in Figure 2a, there was no significant lattice stripe of N-CQDs, which indicated that the prepared N-CQDs were amorphous structures. As can be seen from the FT-IR spectra (Figure 2b), the functional groups of N-CQDs were mainly amine (3421 cm^−1^), methylene (2895, 2965 cm^−1^), and amide carbonyl (1716 cm^−1^), and the chemical bonds were C=C (1458, 1507 cm^−1^), C–N (1410 cm^-1^), aromatic C–NH (1271 cm^−1^), and C–O (1097 cm^−1^). XPS measurements were carried out to probe the N atoms in the N-CQDs. XPS full-scan spectra showed three peaks around 284.8, 399.3, and 532 eV that were ascribed to carbon, nitrogen, and oxygen, respectively (Figure 2c). As shown in Figure 2c,d, there was no significant difference in the concentrations of N1s at depths of 10, 20, and 30 nm from the N-CQDs surface. This indicates that the internal structure of the prepared N-CQDs was uniform. The content of N was about 16% (N/C atomic ratio is about 20.0%), which is much higher than that of various N-doped graphene-based materials (0.3–8.3%) [43,44,45,46]. Elemental analysis indicated that the prepared N-CQDs contained about 19% of nitrogen element.

### 3.2. Characterization of Fluorescence Quantum Yield and Fluorescence Lifetime

**Figure 3:**
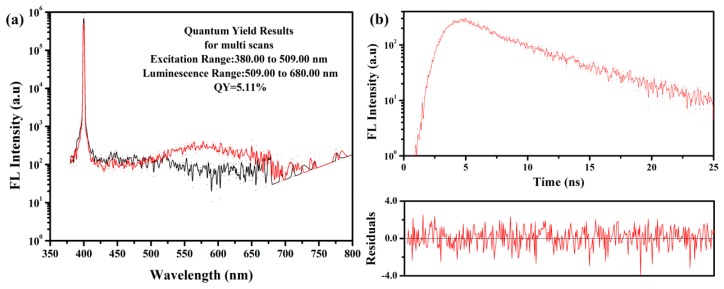
Fluorescence characteristics of N-CQDs. (**a**) Fluorescence quantum yield of N-CQDs; (**b**) fluorescence lifetime index fitting curve of N-CQDs.

As shown in Figure 3a, the fluorescence quantum yield of the prepared N-CQDs was 5.11%, as determined by the calculation formula (1) in 4.4.

The fluorescence lifetime of N-CQDs was measured by time-correlated single-photon counting (TCSPC). After processing and fitting the measurement data, the fluorescence lifetime was obtained, as shown in Figure 3b. According to the exponential fitting curve in Figure 3b and the data (τ_1_ = 2.1605 ns, τ_2_ = 6.4231 ns, B1 = 96.774 %, B2 = 184.864 %, χ^2^ = 1.068) from fitting the parameters of the fluorescence decay curve, the average weighted lifetime of fluorescence attenuation of the N-CQDs under the excitation of 400 nm was 5.79 ns, as determined by using formula (2) in 4.5, where χ^2^ was 1.068.

### 3.3. Fluorescence Intensity of N-CQDs at Different Dilutions

**Figure 4:**
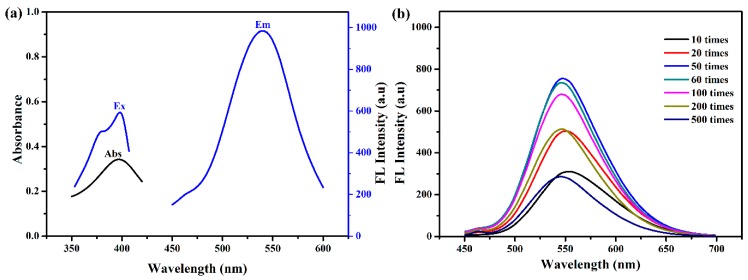
(**a**) UV–vis adsorption and fluorescence spectra of N-CQDs; (**b**) fluorescence intensity of N-CQD solutions.

As shown in Figure 4a, we found an excitation peak around 400 nm. When excited at 400 nm, the N-CQDs exhibited strong photoluminescence (PL) emission centered at 546 nm. The fluorescence intensities of N-CQDs at different dilutions were measured by fluorescence spectrometry (Figure 4b). The fluorescence intensity of N-CQDs diluted 50 times in water was the strongest. Therefore, N-CQDs diluted 50 times in ultrapure water was finally applied for the detection of Ag^+^.

### 3.4. Determination of the Concentration of Silver Ions by N-CQDs

**Figure 5:**
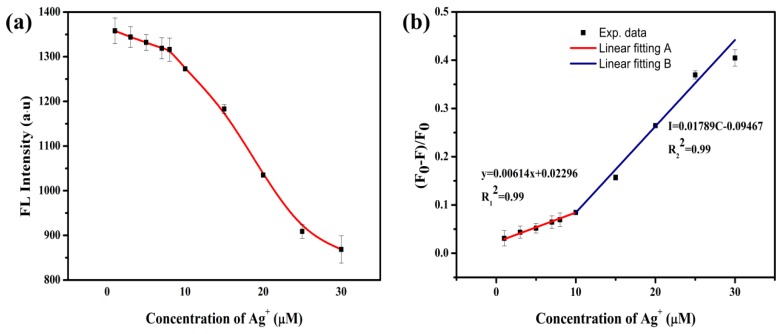
N-CQDs were used for the detection of Ag^+^ in aqueous solution. (**a**) Fluorescence intensity of N-CQDs at different concentrations of Ag^+^; (**b**) fluorescence quenching efficiency of N-CQDs at different concentrations of Ag^+^. In the figure, F_0_ is the fluorescence intensity of N-CQDs; F is the fluorescence intensity of N-CQDs when different concentrations of Ag^+^ are added; (F_0_ − F)/F_0_ is the fluorescence quenching efficiency of N-CQDs.

Various concentrations of Ag^+^ (0–30 μM) were added to the N-CQDs (pH = 7) to explore the performance of N-CQDs in the detection of Ag^+^ in aqueous solution. It can be seen in Figure 5a that the enhancement of the fluorescence quenching effect of N-CQDs can be attributed to the increase of silver ion concentration. As shown in Figure 5b, as the Ag^+^ increased from 0 to 30 μM, the fluorescence quenching efficiency gradually increased. With the increase of Ag^+^ concentration, the quenching efficiency of N-CQDs became increasingly higher, and the maximum efficiency was as high as 40%. The calibration curve of the quenching efficiency depending on Ag^+^ concentrations of 0–10 μM and 10–30 μM can be expressed as y = 0.00614x + 0.02296, with correlation coefficient (R^2^) = 0.99, and I = 0.01789C − 0.09467. The experimental data showed that the fluorescence quenching efficiency of N-CQDs in the presence of Ag^+^ showed a linear relationship with silver ion concentrations of 0–10 μM and 10–30 μM.

### 3.5. Selective Detection of Silver Ions by N-CQDs

**Figure 6:**
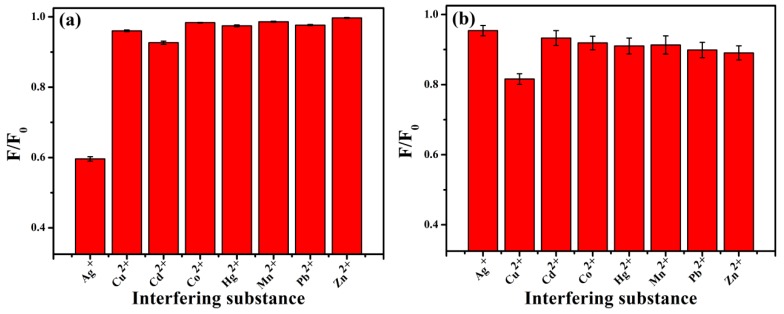
Selectivity of two kinds of quantum dots for different metal ions. F_0_ is the fluorescence intensity of the quantum dots; F is the fluorescence intensity when metal ions are added; F/F_0_ is the fluorescence quenching efficiency of the quantum dots. (**a**) Selectivity of N-CQDs for different metal ions (25 μM); (**b**) selectivity of cadmium selenide (CdSe) quantum dots for different metal ions (25 μM).

As shown in Figure 6a, there was no significant effect on the fluorescence intensity of N-CQD after adding Hg^2+^, Cu^2+^, Cd^2+^, Cd^2+^, Co^2+^, Zn^2+^, or Mn^2+^. Ag^+^ exerted a significant fluorescence quenching effect on N-CQDs, indicating that N-CQDs had a good selectivity for Ag^+^. This phenomenon may be due to the coordination reaction between Ag^+^ and N–H. The types of bonds between Ag^+^ and N-CQDs may be coordination interactions between Ag^+^ and C–N groups. Figure 6b shows that CdSe quantum dots had higher selectivity for copper ions than silver ions.

### 3.6. Detection of Ag^+^ in Food Packaging Materials

The results of the quantitative detection of silver ions in a food packaging sample by the N-CQDs detection method and the Chinese standard method are shown in Table 1.

As shown in Table 1, the concentration of Ag^+^ in the sample was determined to be 1.352 mg/L by the Chinese standard method GB11907-89, corresponding to 22.10 mg/kg. The content of harmful heavy metal ions in packaging materials should not exceed 100 mg/kg according to Chinese food safety regulations, so the Ag^+^ content in this sample did not exceed the standard limit.

The concentration of Ag^+^ in the sample was 1.442 mg/L according to the N-CQDs detection method. As shown in Table 1, the comparison between the results of the N-CQDs detection method and the Chinese standard method GB 11907-89 indicates that the relative error was 6.24%, and the test result was reliable. Food contact materials tend to be contaminated by heavy metals such as lead [47] and cadmium [48] during processing, and the presence of these heavy metal ions causes a certain degree of fluorescence quenching of N-CQDs. Compared with the national standard method (GB 11907-89), the N-CQDs method may have a tendency to produce false positive results. In the experiment shown, the reason may be that the tested food packaging material contained heavy metals such as lead and cadmium.

## 4. Conclusions

In conclusion, N-CQDs with yellow-green fluorescence can be easily prepared through a solvent-thermal reaction. The characterization of the prepared N-CQDs showed that N-CQDs contained various functional groups and chemical bonds that related to N, and their structure was amorphous. Meanwhile, the prepared N-CQDs showed a considerable QY (5.11%) and high chemical and optical stability. More importantly, N-CQDs showed good selectivity for and sensitivity to Ag^+^ at the concentrations of 0–10 μM and 10–30 μM, respectively. N-CQDs were used to detect the content of Ag^+^ in food packaging material. The results showed that the relative error of the N-CQDs detection method with respect to the Chinese standard method was 6.24%, which means the test results of the N-CQDs detection method were reliable. The surface state of N-CQDs can be influenced by Ag^+^ ions. When N-CQDs were free in aqueous solution, they showed strong fluorescence intensity. However, in the presence of silver ions, the fluorescence of N-CQDs was significantly quenched by a charge transfer process. To sum up, the N-CQDs detection method is expected to be useful in the detection of Ag^+^ in food packaging materials.

## Figures and Tables

**Table 1:** Results of the measurement of Ag^+^ concentration in food packaging material.

Methods	Ag^+^ (mg/L)	Linear Range (μM)	Limit of Detection(LOD) (μM)	Relative Error (%)
N-CQDs detection method	1.442 (± 3.8 × 10^−2^)	0–10	1.19	6.24
		10–30	0.66	
Chinese standard method GB 11907-89	1.352 (± 1.8 × 10^−3^)	0–30	0.49	

## References

[1] Matsumura Y., Yoshikata K., Kunisaki S., Tsuchido T. (2003). Mode of bactericidal action of silver zeolite and its comparison with that of silver nitrate. Appl. Environ. Microbiol..

[2] Juan S.M., Segura E.L., Cazzulo J.J. (1979). Inhibition of the NADP-linked glutamate dehydrogenase from Trypanosoma cruzi by silver nitrate. Experientia.

[3] Bragg P.D., Rainnie D.J. (1974). The effect of silver ions on the respiratory chain of *Escherichia coli*. Can. J. Microb..

[4] Feng Q.L., Wu J., Chen G.Q., Cui F.Z., Kim T.N., Kim J.O. (2000). A mechanistic study of the antibacterial effect of silver ions on *Escherichia coli* and Staphylococcus aureus. J. Biomed. Mater. Res..

[5] Nover L., Scharf K.D., Neumann D. (1983). Formation of cytoplasmic heat shock granules in tomato cell cultures and leaves. Mol. Cell. Biol..

[6] Emamifar A., Kadivar M., Shahedi M., Soleimanian-Zad S. (2010). Evaluation of nanocomposite packaging containing Ag and ZnO on shelf life of fresh orange juice. Innov. Food Sci. Emerg. Technol..

[7] Yang F.M., Li H.M., Li F., Xin Z.H., Zhao L.Y., Zheng Y.H., Hu Q.H. (2010). Effect of Nano-Packing on Preservation Quality of Fresh Strawberry (Fragaria ananassa Duch. cv Fengxiang) during Storage at 4 degrees C. J. Food Sci..

[8] Youssef A.M., Abdel-Aziz M.S. (2013). Preparation of Polystyrene Nanocomposites Based on Silver Nanoparticles Using Marine Bacterium for Packaging. Polym.-Plast. Technol. Eng..

[9] Fernandez A., Picouet P., Lloret E. (2010). Reduction of the Spoilage-Related Microflora in Absorbent Pads by Silver Nanotechnology during Modified Atmosphere Packaging of Beef Meat. J. Food Prot..

[10] Incoronato A.L., Conte A., Buonocore G.G., Del Nobile M.A. (2011). Agar hydrogel with silver nanoparticles to prolong the shelf life of Fior di Latte cheese. J. Dairy Sci..

[11] Mastromatteo M., Conte A., Lucera A., Saccotelli M.A., Buonocore G.G., Zambrini A.V., Del Nobile M.A. (2015). Packaging solutions to prolong the shelf life of Fiordilatte cheese: Bio-based nanocomposite coating and modified atmosphere packaging. LWT-Food Sci. Technol..

[12] Takenaka S., Karg E., Roth C., Schulz H., Ziesenis A., Heinzmann U., Schramel P., Heyder J. (2001). Pulmonary and systemic distribution of inhaled ultrafine silver particles in rats. Environ. Health Perspect..

[13] Sung J.H., Ji J.H., Yoon J.U., Kim D.S., Song M.Y., Jeong J., Han B.S., Han J.H., Chung Y.H., Kim J. (2008). Lung function changes in Sprague-Dawley rats after prolonged inhalation exposure to silver nanoparticles. Inhal. Toxicol..

[14] Lee H.Y., Choi Y.J., Jung E.J., Yin H.Q., Kwon J.T., Kim J.E., Im H.T., Cho M.H., Kim J.H., Kim H.Y. (2010). Genomics-based screening of differentially expressed genes in the brains of mice exposed to silver nanoparticles via inhalation. J. Nanopart. Res..

[15] Tang J.L., Xiong L., Wang S., Wang J.Y., Liu L., Li J.G., Yuan F.Q., Xi T.F. (2009). Distribution, Translocation and Accumulation of Silver Nanoparticles in Rats. J. Nanosci. Nanotechnol..

[16] Foldbjerg R., Olesen P., Hougaard M., Dang D.A., Hoffmann H.J., Autrup H. (2009). PVP-coated silver nanoparticles and silver ions induce reactive oxygen species, apoptosis and necrosis in THP-1 monocytes. Toxicol. Lett..

[17] Nishanth R.P., Jyotsna R.G., Schlager J.J., Hussain S.M., Reddanna P. (2011). Inflammatory responses of RAW 264.7 macrophages upon exposure to nanoparticles: Role of ROS-NF kappa B signaling pathway. Nanotoxicology.

[18] Piao M.J., Kang K.A., Lee I.K., Kim H.S., Kim S., Choi J.Y., Choi J., Hyun J.W. (2011). Silver nanoparticles induce oxidative cell damage in human liver cells through inhibition of reduced glutathione and induction of mitochondria-involved apoptosis. Toxicol. Lett..

[19] Carlson C., Hussain S.M., Schrand A.M., Braydich-Stolle L.K., Hess K.L., Jones R.L., Schlager J.J. (2008). Unique Cellular Interaction of Silver Nanoparticles: Size-Dependent Generation of Reactive Oxygen Species. J. Phys. Chem. B.

[20] Braydich-Stolle L., Hussain S., Schlager J.J., Hofmann M.C. (2005). In vitro cytotoxicity of nanoparticles in mammalian germline stem cells. Toxicol. Sci..

[21] Gupta A., Maynes M., Silver S. (1998). Effects of halides on plasmid-mediated silver resistance in *Escherichia coli*. Appl. Environ. Microbiol..

[22] Li Y.L., Leng Y.M., Zhang Y.J., Li T.H., Shen Z.Y., Wu A.G. (2014). A new simple and reliable Hg 2+ detection system based on anti-aggregation of unmodified gold nanoparticles in the presence of O -phenylenediamine. Sens. Actuators B Chem..

[23] Barriada J.L., Tappin A.D., Evans E.H., Achterberg E.P. (2007). Dissolved silver measurements in seawater. TrAC Trends Anal. Chem..

[24] Ghaedi M., Shokrollahi A., Niknam K., Niknam E., Najibi A., Soylak M. (2009). Cloud point extraction and flame atomic absorption spectrometric determination of cadmium(II), lead(II), palladium(II) and silver(I) in environmental samples. J. Hazard. Mater..

[25] Hartmann G., Hutterer C., Schuster M. (2013). Ultra-trace determination of silver nanoparticles in water samples using cloud point extraction and ETAAS. J. Anal. At. Spectrom..

[26] Duong T.Q., Kim J.S. (2010). Fluoro- and Chromogenic Chemodosimeters for Heavy Metal Ion Detection in Solution and Biospecimens. Chem. Rev..

[27] Xu Z., Kim S.K., Yoon J. (2010). Revisit to imidazolium receptors for the recognition of anions: Highlighted research during 2006–2009. Chem. Soc. Rev..

[28] Chen X.Q., Zhou G.D., Peng X.J., Yoon J. (2012). Biosensors and chemosensors based on the optical responses of polydiacetylenes. Chem. Soc. Rev..

[29] Liu H.F., Sun Y.Q., Yang J., Hu Y.L., Yang R., Li Z.H., Qu L.B., Lin Y.H. (2019). High performance fluorescence biosensing of cysteine in human serum with superior specificity based on carbon dots and cobalt-derived recognition. Sens. Actuator B-Chem..

[30] Gupta V., Chaudhary N., Srivastava R., Sharma G.D., Bhardwaj R., Chand S. (2011). Luminscent Graphene Quantum Dots for Organic Photovoltaic Devices. J. Am. Chem. Soc..

[31] Yan X., Cui X., Li B.S., Li L.S. (2010). Large, Solution-Processable Graphene Quantum Dots as Light Absorbers for Photovoltaics. Nano Lett..

[32] Zhuo S.J., Shao M.W., Lee S.T. (2012). Upconversion and Downconversion Fluorescent Graphene Quantum Dots: Ultrasonic Preparation and Photocatalysis. ACS Nano.

[33] Liu H.F., Sun Y.Q., Li Z.H., Yang J., Aryee A.A., Qu L.B., Du D., Lin Y.H. (2019). Lysosome-targeted carbon dots for ratiometric imaging of formaldehyde in living cells. Nanoscale.

[34] Cao L., Wang X., Meziani M.J., Lu F.S., Wang H.F., Luo P.J.G., Lin Y., Harruff B.A., Veca L.M., Murray D. (2007). Carbon dots for multiphoton bioimaging. J. Am. Chem. Soc..

[35] Yang S.T., Cao L., Luo P.G.J., Lu F.S., Wang X., Wang H.F., Meziani M.J., Liu Y.F., Qi G., Sun Y.P. (2009). Carbon Dots for Optical Imaging in vivo. J. Am. Chem. Soc..

[36] Dong Y.Q., Pang H.C., Yang H.B., Guo C.X., Shao J.W., Chi Y.W., Li C.M., Yu T. (2013). Carbon-Based Dots Co-doped with Nitrogen and Sulfur for High Quantum Yield and Excitation-Independent Emission. Angew. Chem.-Int. Edit..

[37] Chandra S., Patra P., Pathan S.H., Roy S., Mitra S., Layek A., Bhar R., Pramanik P., Goswami A. (2013). Luminescent S-doped carbon dots: An emergent architecture for multimodal applications. J. Mater. Chem. B.

[38] Zhang P., Li W.C., Zhai X.Y., Liu C.J., Dai L.M., Liu W.G. (2012). A facile and versatile approach to biocompatible “fluorescent polymers” from polymerizable carbon nanodots. Chem. Commun..

[39] Zhang Y.Q., Ma D.K., Zhuang Y., Zhang X., Chen W., Hong L.L., Yan Q.X., Yu K., Huang S.M. (2012). One-pot synthesis of N-doped carbon dots with tunable luminescence properties. J. Mater. Chem..

[40] Sun H.Z., Zhang F., Wei H.T., Yang B. (2013). The effects of composition and surface chemistry on the toxicity of quantum dots. J. Mater. Chem. B.

[41] Guo B.D., Liu Q.A., Chen E.D., Zhu H.W., Fang L.A., Gong J.R. (2010). Controllable N-Doping of Graphene. Nano Lett..

[42] Sajwan R.K., Bagbi Y., Sharma P., Solanki P.R. (2017). L-cysteine and 3-mercaptopropionic acid capped cadmium selenide quantum dots based metal ion probes. J. Lumin..

[43] Tang L.B., Ji R.B., Li X.M., Bai G.X., Liu C.P., Hao J.H., Lin J.Y., Jiang H.X., Teng K.S., Yang Z.B. (2014). Deep Ultraviolet to Near-Infrared Emission and Photoresponse in Layered N-Doped Graphene Quantum Dots. ACS Nano.

[44] Li M., Wu W.B., Ren W.C., Cheng H.M., Tang N.J., Zhong W., Du Y.W. (2012). Synthesis and upconversion luminescence of N-doped graphene quantum dots. Appl. Phys. Lett..

[45] Panchokarla L.S., Subrahmanyam K.S., Saha S.K., Govindaraj A., Krishnamurthy H.R., Waghmare U.V., Rao C.N.R. (2009). Synthesis, Structure, and Properties of Boron- and Nitrogen-Doped Graphene. Adv. Mater..

[46] Zhang C.H., Fu L., Liu N., Liu M.H., Wang Y.Y., Liu Z.F. (2011). Synthesis of Nitrogen-Doped Graphene Using Embedded Carbon and Nitrogen Sources. Adv. Mater..

[47] Goodlaxson B., Curtzwiler G., Vorst K. (2018). Evaluation of methods for determining heavy metal content in polyethylene terephthalate food packaging. J. Plast. Film Sheeting.

[48] Kim K.C., Park Y.B., Lee M.J., Kim J.B., Huh J.W., Kim D.H., Lee J.B., Kim J.C. (2008). Levels of heavy metals in candy packages and candies likely to be consumed by small children. Food Res. Int..

